# SARS-CoV-2 infection among employees working from home and on site: An occupational study in Switzerland

**DOI:** 10.3389/fpubh.2022.980482

**Published:** 2022-09-16

**Authors:** Alexia Schmid, Daniela Anker, Julie Dubois, Isabelle Bureau-Franz, Nathalie Piccardi, Sara Colombo Mottaz, Stéphane Cullati, Arnaud Chiolero, Pierre-Yves Rodondi

**Affiliations:** ^1^Institute of Family Medicine, Faculty of Science and Medicine, University of Fribourg, Fribourg, Switzerland; ^2^Population Health Laboratory ^#^PopHealthLab, Faculty of Science and Medicine, University of Fribourg, Fribourg, Switzerland; ^3^Nestlé Research, Lausanne, Switzerland; ^4^Department of Readaptation and Geriatrics, University of Geneva, Geneva, Switzerland; ^5^Institute of Primary Health Care, Faculty of Medicine (BIHAM), University of Bern, Bern, Switzerland; ^6^School of Population and Global Health, Faculty of Medicine and Health Sciences, McGill University, Montréal, QC, Canada

**Keywords:** occupational health, SARS-CoV-2 infection (COVID-19), COVID-19, workplace, employees, work from home

## Abstract

During the COVID-19 pandemic, many companies implemented working from home to mitigate the spread of the disease among their employees. Using data from *Corona Immunitas Nestlé*, a seroepidemiological study conducted among employees from two Nestlé sites in Switzerland, we aimed to investigate whether there was a difference in SARS-CoV-2 infection rates between employees working most of the time from home and employees mobilized in a workplace equipped with a specialized occupational safety unit and strict sanitary measures. We also investigated whether this association was modified by household size, living with children, vulnerability, worries about an infection, and worries about adverse health consequences if infected. Data were collected between 8 December 2020, and 11 February 2021. Previous SARS-CoV-2 infections were ascertained by the presence of anti-SARS-CoV-2 IgG antibodies in the blood. Of the 425 employees included (53% women; mean age 42 years ranging between 21 and 64 years), 37% worked most of the time from home in 2020 and 16% had been infected with SARS-CoV-2. Participants who worked most of the time from home in 2020 had slightly higher odds of being infected with SARS-CoV-2 compared to participants who never or only sometimes worked from home (adjusted OR 1.29, 95% CI 0.73–2.27). The association was stronger in participants living alone or with one other person (adjusted OR 2.62, 95% CI 1.13–6.25). Among participants living with two or more other persons (adjusted OR 0.66, 95% CI 0.30–1.39) and among vulnerable participants (adjusted OR 0.53, 95% CI 0.13–1.93), working from home tended to be associated with lower odds of infection. In conclusion, in a context of strict sanitary measures implemented in the workplace, employees working from home did not seem to be at lower risk of infection compared to those working on site, especially if living alone or with one other person.

## Introduction

The Coronavirus disease (COVID-19) pandemic, caused by the severe acute respiratory syndrome coronavirus 2 (SARS-CoV-2), had an immense impact on work environments with the implementation of sanitary measures and restrictions at workplaces. Many companies implemented working from home as well as strict sanitary measures in the workplace to mitigate the spread of SARS-CoV-2 among their employees (1–4). Compared to working from home, working on site may place employees at increased risk for SARS-CoV-2 infection due to potential exposure to infected individuals on the way to work and in the workplace, e.g., through potentially close proximity in enclosed spaces (2, 3, 5). The risk of infection, however, also depends on the exposure to SARS-CoV-2 outside of the workplace, which is probably related to the social contacts a person had. Indeed, a population-based study conducted among the Swiss population suggested that living with children was associated with SARS-CoV-2 seropositivity (6), and in a case-control study by Galmiche et al., a higher risk of infection was reported in larger households and in households with children (7). A meta-analysis by Madewell et al. indicated that households are important sites contributing to SARS-CoV-2 transmission (8).

Therefore, we aimed to investigate whether employees working from home and employees mobilized in a workplace with strict sanitary measures had different SARS-CoV-2 infection rates. Further, we investigated whether this difference would change depending on employees' social contacts, i.e., household size and number of children, as well as their level of worries about an infection and their vulnerability [defined according to the criteria of the Federal Office of Public Health in Switzerland (9)].

Using data from a seroepidemiological study conducted among employees from two Nestlé sites with strict sanitary measures implemented in the workplace, the specific objectives were to investigate:

- The proportion of employees having been infected with SARS-CoV-2.
- Whether SARS-CoV-2 infection rates differed between employees working from home and those on site.
- Whether people living in households with fewer people and without children had fewer infections when working from home.
- Whether vulnerable people or people worried about an infection had fewer infections when working from home.

## Materials and methods

This manuscript was written using the *Strengthening the Reporting of Observational Studies in Epidemiology (STROBE) statement: guidelines for reporting observational studies* (10).

### Study design and participants

**Figure 1:**
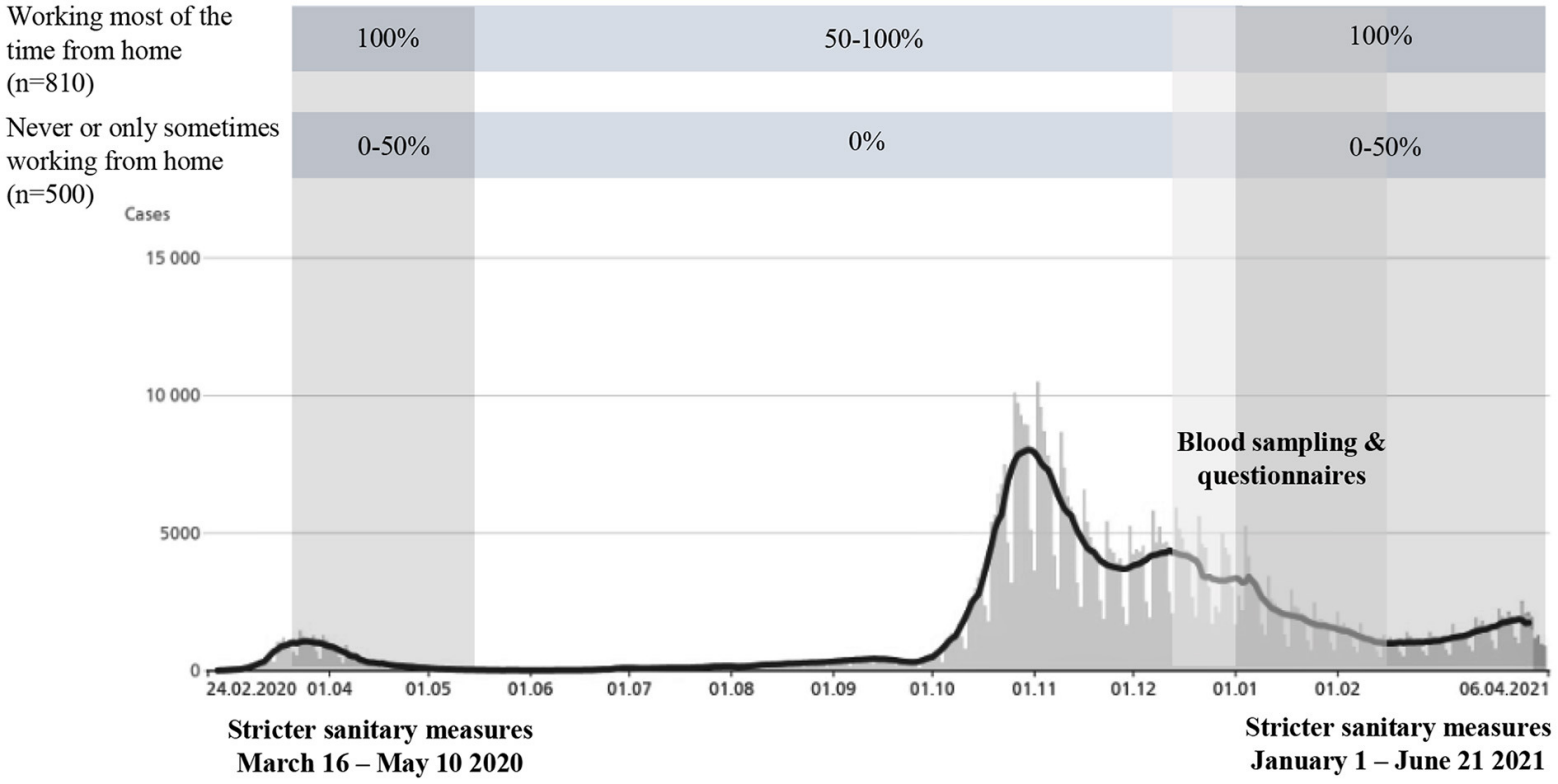
Time percentage of work from home among employees in the Nestlé company classified into two exposure groups (*working most of the time from home* and *never or only sometimes working from home*) over time and in relation to the COVID-19 pandemic and the *Corona Immunitas Nestlé* study. The graph shows the laboratory-confirmed cases in Switzerland from 24.02.2020 to 06.04.2021, in absolute case numbers. It is important to note that the number of tests performed has increased over time, since only hospitalized at-risk persons were tested in the beginning of the pandemic (34). *n* = number of employees invited to participate in the study. Figure adapted from Schmid et al. (35).

We used data from the study *Corona Immunitas Nestlé*, which was part of *Corona Immunitas (https://www.corona-immunitas.ch/en/)*, a Swiss national research program of seroepidemiological studies conducted during the COVID-19 pandemic and coordinated by the Swiss School of Public Health (SSPH+; https://ssphplus.ch/) (11). *Corona Immunitas Nestlé* was conducted among employees from two Nestlé sites in Switzerland, a research center (Lausanne, *n* = 920) and a factory (Romont, *n* = 390). These sites were selected because the scope of their activities required the presence of employees in the workplace. All employees aged 18 years and older working at the two specific sites were invited by e-mail to participate (*n*_*tot*_ = 1,310). Study visits were carried out by Nestlé trained medical staff on work sites. Venous blood samples and questionnaire data were collected between December 8, 2020, i.e., during the descending phase of the second pandemic wave in Switzerland, and February 11, 2021, i.e., at the lowest case number between the second and third pandemic waves (Figure 1). At the time of this study, no vaccine against SARS-CoV-2 was available in Switzerland, and therefore, antibody positivity among participants was related to previous infection only.

Switzerland has sought to find a balance between controlling the propagation of COVID-19 and maintaining a certain normality in social and economic life. A complete lockdown consisting of staying home on legal orders has never occurred. Sanitary measures were most restrictive during the first pandemic wave from March to May 2020, when schools, most businesses, and facilities were closed. Companies were invited to have their employees working from home, whenever possible. The second wave, at the end of October 2020, resulted in a more heterogeneous response with fewer restrictions, with each canton having the role of making decisions (12, 13).

Since the beginning of the pandemic in Switzerland (March 2020), the two Nestlé sites have implemented strict sanitary measures in the workplace as well as work from home for employees whose work could be performed remotely. Implemented sanitary measures were for example: temperature control at the entry of the buildings, mandatory wearing of sanitary masks, environmental adjustments to enable physical distance, and surveillance measures, e.g. regular COVID-19 testing. In addition, employees were divided into groups that could not be in the workplace at the same time to further reduce the risks of virus transmission. These measures were maintained during the whole duration of this study, however, with stricter measures implemented during the first wave of the pandemic in spring 2020, from March 16 to May 10, and again starting January 1, 2021. We distinguished two groups of employees subject to different measures (Figure 1). At the research center, 80% of employees (*n* = 720) worked entirely from home in spring 2020, and then partially on site until December 2020, and 20% (*n* = 200) continued to work on site to ensure business continuity. At the factory, 75% of employees (*n* = 300) worked part-time on site in spring 2020 and fulltime until December 2020 due to their involvement in the factory production. The remaining employees were those who only performed administrative tasks (25%, *n* = 90) and worked fulltime from home since the beginning of the pandemic and throughout the period covered by this study.

### Serology analysis

Venous blood samples (5 ml per participant) were collected and transported to the Lausanne University Hospital (CHUV) the same day. Blood analyses were performed using the SenASTrIS (Sensitive Anti-SARS-CoV-2 Spike Trimer Immunoglobulin Serological) test developed by the CHUV, the Swiss Federal Institute of Technology in Lausanne (EPFL) and the Swiss Vaccine Center (14). The Luminex binding assay measured binding of IgG antibodies to the trimeric SARS-CoV-2 S-protein. According to the test developers, participants were positive for SARS-CoV-2 antibodies if the antibody response was equal or above the cut-off of six, negative below the cut-off of four, and indeterminate in between. The specificity and sensitivity of 99.7 and 96.6%, respectively, from 15 days after a SARS-CoV-2 infection were defined on a population-based sample, hence limiting spectrum bias (11, 15).

### Questionnaire

Questionnaires were completed online by the participants during the study visit. The *Corona Immunitas Nestlé* questionnaire was based on the *Corona Immunitas* baseline questionnaire (available in the published protocol (11)), and included information about health status, sociodemographic data, household characteristics and worries about the coronavirus situation. A specific section was added for this study to assess working from home.

### Variables of interest

The exposure of interest was working from home, ascertained by the question “have you been concerned by working from home during the first wave of the pandemic (March 16 to May 10)?” to which participants replied either “yes, most of the time,” “yes, but only sometimes,” or “no.” It allowed us to determine the working conditions of the participants in spring 2020 and from there, we assumed how the participants worked during the rest of the year given the above-described work organization implemented by the two Nestlé sites. Two exposure groups were defined: (1) participants who were *most of the time working from home in 2020;* we considered that participants who reported working most of the time from home during spring 2020 also worked either full time or minimum 50% from home until winter 2020-2021, (2) participants who were *never or only sometimes working from home in 2020*; we considered that participants who reported never or only sometimes working from home during spring 2020 fully worked on site until winter 2020–2021 (Figure 1). Although strongly mobilized on site, we considered the second group to have a moderate exposure to SARS-CoV-2 in the workplace because the company implemented strict sanitary measures to reduce SARS-CoV-2 exposure.

The outcome variable was the presence of IgG antibodies against SARS-CoV-2 in the blood considered as a proxy for having been infected with the virus at least once since the beginning of the pandemic. We considered that participants with a positive serology result had been infected, and those with a negative or indeterminate result had not been infected since the beginning of the pandemic. At the time of this study, vaccination against SARS-CoV-2 was not yet available in Switzerland.

Potential confounders were identified using a directed acyclic graph (Supplementary Figure 1) and included age, gender, education, work site and vulnerability. Potential effect modifiers of the association between working from home and SARS-CoV-2 infection included household size, living with children, vulnerability, worries about an infection, and worries about adverse health consequences if infected. Age was grouped into three categories *18*–*34; 35*–*49; 50*–*65* years. Education was grouped into a *basic educational level* (mandatory education, apprenticeship or Matura) and an *advanced educational level* (technical college, university or polytechnic).

Participants were considered vulnerable, i.e., having a condition that increases their risk of developing a severe form of COVID-19, in case of a self-reported pregnancy, obesity, or a diagnosis of cancer, diabetes, hypertension, cardiovascular disease, chronic respiratory disease or a disease that weaken the immune system, or the intake of a treatment that weaken the immune system (as defined by the Federal Office of Public Health in Switzerland (9)). Obesity was defined by a BMI above 30 kg/m^2^, calculated from self-reported weight and height. A participant was considered *vulnerable* when concerned by at least one vulnerability criterion.

Household size was defined by the number of people currently living in the same household as the participant, and was grouped into two categories: *living alone or with one other person* in the household or *living with* ≥ *2 other persons* in the household. Participants were classified as *living with children* if one or more of their household members were under the age of 18. Worries about being infected and about adverse health consequences if infected were ascertained by questionnaire in which participants were asked to what extent they were worried about contracting the virus or about the consequences of an infection on their own health and they could answer “not at all,” “a little,” “moderately,” “very,” or “extremely.” Two categories, for each variable, were defined: those, who were *very to extremely worried* and those, who were *not at all to moderately worried* about an infection or adverse health consequences if infected.

### Statistical analysis

We modeled the association between working from home and SARS-CoV-2 infection through logistic regression analyses. Three sets of models were fitted: model (1) unadjusted, model (2) adjusted for age and gender, and model (3) adjusted for age, gender, education, work site, and vulnerability. Results were reported as odds ratios and 95% confidence interval in tables and visualized on the logarithmic scale in plots. To evaluate effect modification, we modeled the association between working from home and SARS-CoV-2 infection across strata of the potential effect modifier under consideration.

In sensitivity analysis, we performed the same analyses excluding participants who *only sometimes worked from home*, hence only those who reported *never working from home* and those who reported *most of the time working from home* during spring 2020 were considered. Missing data for variables used in the analyses are reported in Table 1. Statistical analyses were performed using the software R (version 4.1.0).

**Table 1:** Characteristics of study participants overall and of those infected with SARS-CoV-2^a^.

	***N* (%)**	**Infected with SARS-CoV-2 (%)**
Total	425 (100)	66 (16)
**Gender**		
Women	224 (53)	32 (14)
Men	201 (47)	34 (17)
**Age group, years**		
18–34	103 (24)	24 (23)
35–49	214 (50)	25 (12)
50–65	108 (25)	17 (16)
**Education level** ^**b**^		
Basic education	116 (27)	20 (17)
Advanced education	309 (73)	46 (15)
**Household size**		
Living alone or with one other person	179 (42)	29 (16)
Living with ≥2 other persons	246 (58)	37 (15)
**Children in household**		
Living without children	230 (54)	39 (17)
Living with ≥1 child	195 (46)	27 (14)
**Vulnerability criteria** ^**c**^		
None	337 (79)	53 (16)
≥1	88 (21)	13 (15)
**PCR test** ^**d**^		
None	145 (34)	14 (10)
Positive result	27 (6)	24 (89)
Negative result	244 (57)	27 (11)
Unknown result	9 (2)	1 (11)
**Worried about being infected**		
Not at all to moderately	352 (83)	54 (15)
Very to extremely	73 (17)	12 (16)
**Worried about adverse health**		
**consequences if infected**		
Not at all to moderately	349 (82)	51 (15)
Very to extremely	75 (18)	15 (20)
Missing data	1 (0)	0 (0)
**Nestlé sites**		
Nestlé research center (Lausanne, Vaud)	299 (70)	46 (15)
Nestlé factory (Romont, Fribourg)	126 (30)	20 (16)
**Work from home in 2020**		
Never or sometimes	266 (63)	39 (15)
Most of the time	159 (37)	27 (17)

Results are N (%). All data beside serology, gender and age, are self-reported.

a Participants with positive IgG antibodies to SARS-CoV-2 in the blood are considered to have been infected with the virus at least once since the beginning of the pandemic.

b Basic education includes mandatory education, apprenticeship or Matura. Advanced education includes technical college, university or polytechnic.

c Vulnerability is defined according to the criteria of the Federal Office of Public Health (9).

a Participants were asked whether they had taken a PCR test since the beginning of the pandemic before the study blood test, and about the result of the test. Some PCR tests have been taken shortly before the blood test and the results were therefore still unknown when the participants completed the questionnaire.

## Results

Overall, 1,310 employees from both Nestlé sites were invited and 425 (33%) participated. Characteristics of participants are reported in Table 1. Some 53% of the participants were women and mean age was 42 years (age range between 21 and 64 years). Some 37% worked most of the time from home during 2020 and 63% never or only sometimes worked from home. Overall, 16% had been infected with SARS-CoV-2 by February 2021.

The associations between SARS-CoV-2 infection and work from home are reported in Table 2. Participants working most of the time from home in 2020 had higher odds of being infected with SARS-CoV-2 compared to participants never or only sometimes working from home. The confidence intervals were wide, however, and crossed the null value. Performing similar analyses in the sample without the participants who only sometimes worked from home led to similar results (Supplementary Table 1).

**Table 2:** Association between work from home and SARS-CoV-2^a^ infection assessed through logistic regression (*n* = 425).

	Model 1^*^ OR (95% CI)	Model 2^**^ OR (95% CI)	Model 3^***^ OR (95% CI)
**Work from home**			
Never or sometimes	Ref	Ref	Ref
Most of the time	1.19 (0.69–2.03)	1.22 (0.70–2.11)	1.29 (0.73–2.27)
**Gender**			
Women	**–**	Ref	Ref
Men	**–**	1.32 (0.77–2.28)	1.39 (0.78–2.49)
**Age group, years**			
18–34	–	Ref	Ref
35–49	–	0.43 (0.23–0.79)	0.43 (0.2–0.79)
50–65	–	0.62 (0.31–1.24)	0.58 (0.27–1.19)
**Education** ^**b**^			
Basic education	**–**	**–**	Ref
Advanced education	**–**	**–**	0.75 (0.40–1.45)
**Work site**			
Nestlé research center (Lausanne, Vaud)	**–**	**–**	Ref
Nestlé factory (Romont, Fribourg)	**–**	**–**	0.86 (0.44–1.64)
**Vulnerability**			
None	**–**	**–**	Ref
≥1	**–**	**–**	0.89 (0.44–1.72)

* Unadjusted.

** Adjusted for age and gender.

*** Adjusted for age, gender, education, work site, vulnerability.

OR = odds ratio, 95% CI = 95% confidence interval.

a Participants with positive IgG antibodies to SARS-CoV-2 in the blood are considered to have been infected with the virus at least once since the beginning of the pandemic.

b Basic education includes mandatory education, apprenticeship or Matura. Advanced education includes technical college, university or polytechnic.

**Figure 2:**
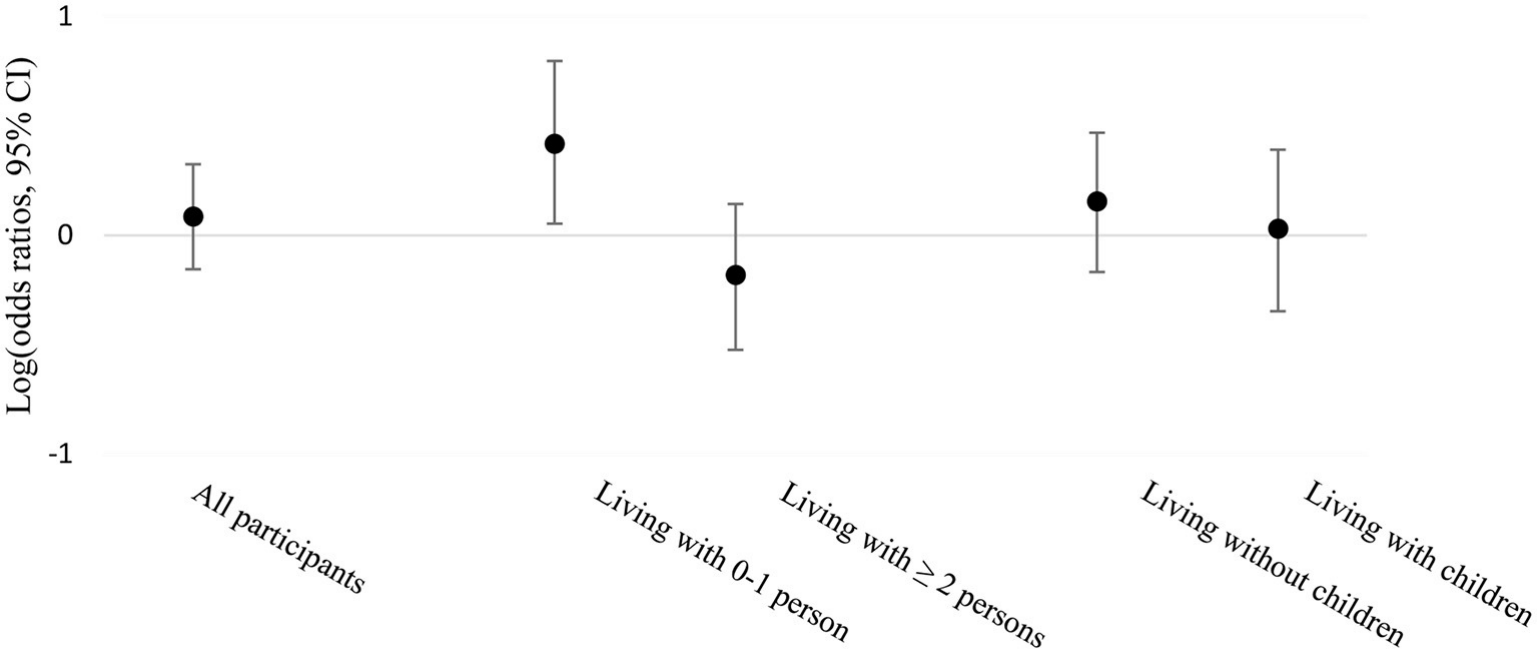
Association between work from home and SARS-CoV-2 infection stratified by household size and living with children or not, displayed on the logarithmic scale of odds ratios and 95% CI.

The associations between SARS-CoV-2 infection and work from home across strata of household size and living with children are reported in Table 3; Figure 2. Among participants living alone or with one other person in the household, those who worked most of the time from home had higher odds of being infected with SARS-CoV-2 compared to those who never or only sometimes worked from home with wide confidence intervals but not crossing the null value (adjusted OR 2.62, 95% CI 1.13–6.25). Among participants living with two or more other persons in the household, the odds of being infected were lower when working most of the time from home compared to those who never or only sometimes worked from home. Therefore, participants living with fewer household members seemed at higher risk of infection when working from home, while those living with more than two people seemed at lower risk. Furthermore, working from home was associated with more infections among participants living without children, whereas no association was observed among participants living with children, although the confidence intervals were wide and crossed the null value.

**Table 3:** Association between work from home and SARS-CoV-2^a^ infection stratified by potential effect modifiers.

	**Unadjusted OR (95% CI)**	Adjusted OR^*^ (95% CI)
**Household size**		
Living alone or with one other person	2.46 (1.10–5.60)	2.62 (1.13–6.25)
Living with ≥2 other persons	0.66 (0.29–1.36)	0.66 (0.30–1.39)
**Children in household**		
Living without children	1.41 (0.69–2.85)	1.43 (0.68–2.94)
Living with ≥1 child	0.99 (0.42–2.24)	1.07 (0.45–2.46)
**Vulnerability criteria** ^**b**^		
None	1.39 (0.76–2.51)	1.44 (0.79–2.63)
≥1	0.63 (0.16–2.13)	0.53 (0.13–1.93)
**Worried about being infected**		
Not at all to moderately	1.15 (0.63–2.06)	1.13 (0.62–2.05)
Very to extremely	1.53 (0.37–5.64)	2.08 (0.46–9.07)
**Worried about adverse health consequences if infected**		
Not at all to moderately	0.96 (0.51–1.76)	0.96 (0.51–1.76)
Very to extremely	0.91 (0.29–2.68)	0.99 (0.30–3.16)

* Adjusted for age and gender.

OR = odds ratio, 95% CI = 95% confidence interval.

a Participants with positive IgG antibodies to SARS-CoV-2 in the blood are considered to have been infected with the virus at least once since the beginning of the pandemic.

b Vulnerability is defined according to the criteria of the Federal Office of Public Health (9).

**Figure 3:**
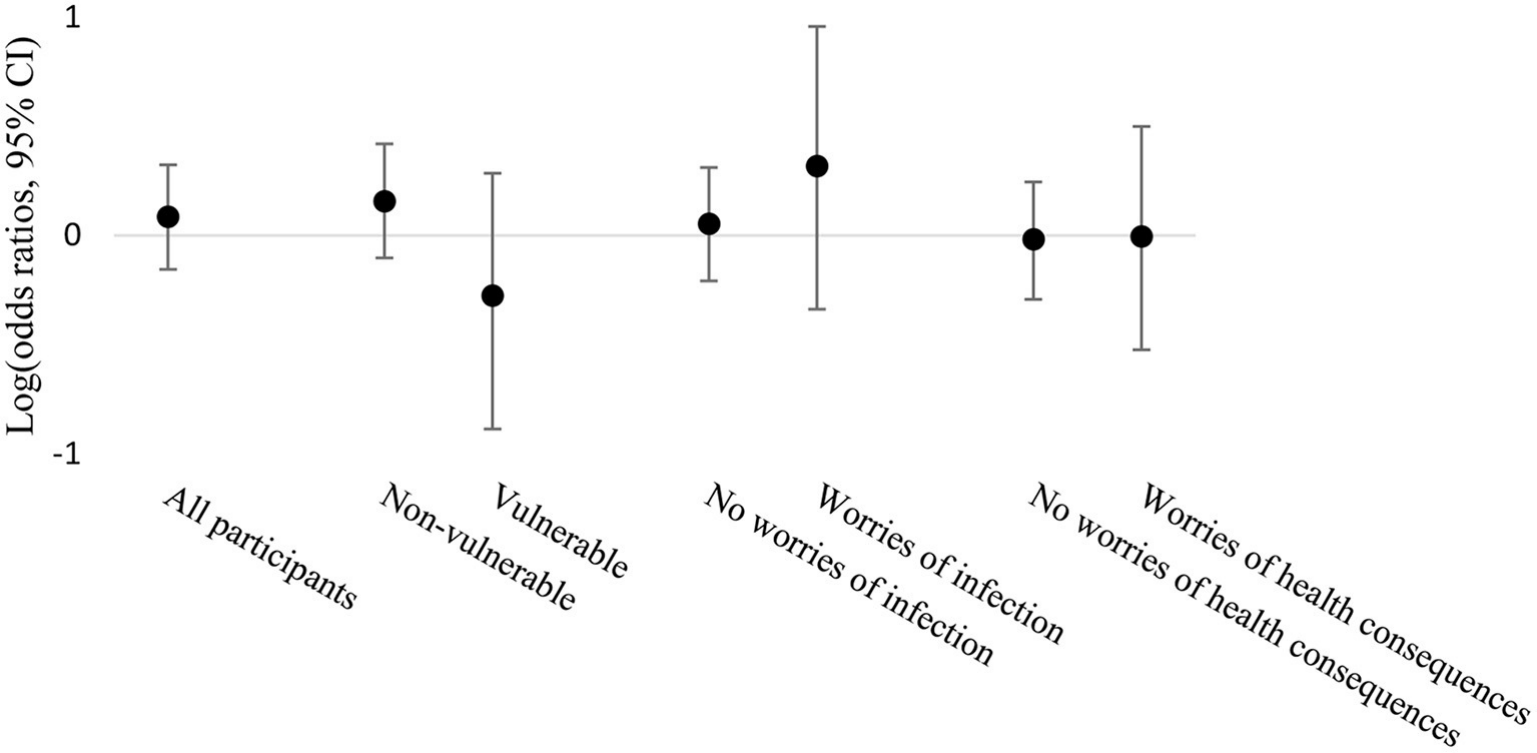
Association between work from home and SARS-CoV-2 infection stratified by vulnerability, worries about being infected and worries about adverse health consequences if infected, displayed on the logarithmic scale of odds ratios and 95% CI.

The associations between SARS-CoV-2 infection and work from home across strata of vulnerability and worries are reported in Table 3; Figure 3. Vulnerable participants tended to have lower odds of being infected when they worked from home compared to when they never or only sometimes worked from home, while non-vulnerable participants tended to have higher odds. While working from home was associated with more infections whatever the worries of being infected were, working from home was not associated with more infections when stratified by worries about adverse health consequences if infected with the virus. One missing value was reported among the variable concerning worries about adverse health consequences if infected and was not used in the analysis. In all these analyses, confidence intervals were wide and crossed the null value. Supplementary Table 2 summarizes the number of participants being infected or not with SARS-CoV-2 in each subgroup of potential effect modifiers.

## Discussion

In this seroepidemiological study, we investigated whether employees working from home in 2020, compared to those working on site with strict sanitary measures, had different SARS-CoV-2 infection rates by February 2021. The sample population included employees from two Nestlé sites based in Switzerland. Our results suggested overall higher odds of being infected with SARS-CoV-2 when working most of the time from home compared to never or only sometimes working from home. This finding suggests that, in a setting with strict sanitary measures implemented in the workplace, which aimed at limiting SARS-CoV-2 transmission, employees mobilized on site were not at an increased risk of SARS-CoV-2 infection compared to those working from home. We observed a stronger association between working from home and SARS-CoV-2 infection in participants living alone or with one other person, while among participants living with two or more other persons and among those vulnerable, working from home tended to be associated with lower odds of being infected.

To our knowledge, only few studies have investigated the relationship between SARS-CoV-2 infection among employees working from home and on site, and none has investigated effect modification on this relationship. Consistent with our findings, in a study conducted among essential workers recruited in various companies in Geneva, Switzerland, Stringhini et al. found that the risk of infection ascertained by the presence of anti-SARS-CoV-2 IgG antibodies between May and September 2020 was higher in participants who were fully working from home in spring 2020 compared to those who were working from home only partially or not at all (RR 1.27, 95% CI 1.03–1.56) (16). On the contrary, in a case-control study by Galmiche et al., whereby PCR-diagnosed COVID-19 cases retrieved from a national database in France were compared to matched controls with no previously suspected SARS-CoV-2 infection, working from home in the 10 days previous to infection or enrollment was associated with a lower risk of SARS-CoV-2 infection (7). Hence, evidence for the relationship between working from home and SARS-CoV-2 infection is currently scarce and contradictory, and differences in study design and setting complicate comparisons.

Our findings from the effect modification analyses show that working most of the time from home did not result in fewer SARS-CoV-2 infections, especially among those with fewer social contacts in the household. It is possible that people, who lived with fewer household members and worked from home were going out more often, for example to fitness centers or bars, which were settings with less strict sanitary measures than in the workplace and which have been shown to increase the risk of infection (7, 17). They may also be less compliant with protective measures because of their reduced responsibility for other household members. Participants living with children may have less non-household contacts overall because they are more likely to stay at home and spend time with their family (18). Our findings, however, support the hypothesis that working most of the time from home would lead to fewer SARS-CoV-2 infections among vulnerable individuals. Vulnerable individuals were advised to stay at home and to take special care to avoid infection to reduce the risk of developing a severe form of COVID-19 (9). Hence, working from home and limiting social contacts may be an additional opportunity for vulnerable individuals to avoid exposure to SARS-CoV-2 and thus reduce the number of infections.

Our study has several limitations. We may underestimate the proportion of participants who were infected. Indeed, there is evidence that following an infection, some people do not develop antibodies (19). In addition, some participants may have been recently infected and not yet developed antibodies. It is also possible that some people who were infected a few months before the data were collected for this study developed antibodies, but that they declined over time (20). Another limitation is that we cannot identify the time of infection. Hence, it is possible that some participants were infected and developed antibodies before any sanitary measures were implemented. As a result, it is more difficult to show a difference in risk between working from home and on site.

While we included up to 33% of all invited employees, our study may be subject to some selection bias. For instance, employees working from home may have had a lower willingness to participate since study visits required to come to the workplace for the blood test, especially as recruitment started shortly before a reinforcement of the sanitary measures from early January 2021. Also, having had a previous infection might have impacted the willingness to participate, although it is hard to tell in which direction. Hence, the willingness to participate could be a collider on which we conditioned our analyses, therefore introducing bias (21).

Furthermore, our study may be subject to some misclassification bias both in the exposure and the outcome. First, we classified participants into two exposure groups based on both self-reported share of work from home during spring 2020 and the work organization implemented by the company, from which we assumed how the participants worked throughout the year. This may have led to random misclassification in the exposure, resulting in estimates biased toward the null value (22). Second, although a highly sensitive and specific SARS-CoV-2 antibody test was used (11, 15), we did not account for antibody waning over time, which may especially concern individuals who were infected early during the pandemic (20). Nevertheless, we assume that this possible misclassification in the outcome is likely to be non-differential with respect to working from home or not, which should not bias the estimate but could increase the variability around the estimates (22).

Finally, the limited number of participants reduced the statistical power of our analyses, especially for the ones conducted in strata of the sample. Most of the estimates we found had wide confidence intervals crossing the null value, hence it is possible that individuals working from home did not truly have higher odds of being infected. Overall, we did not find a clear signal, which may be due to misclassification in both the exposure and the outcome as well as the low sample size. Ideally, this study should be repeated in a larger sample.

Our study has several strengths. This study is one of the few conducted at a company outside of the healthcare sector with an implementation of work from home when possible and strict sanitary measures implemented on site. The main strength is the use of the presence of antibodies in a population-based sample to ascertain the cumulative number of SARS-CoV-2 infections instead of PCR-based case finding. Indeed, the latter only detects acute infections (23) and is conditioned by the testing strategy in place, whereby for instance asymptomatic cases are mostly underreported (24). On the contrary, antibody testing on a population-based sample also detects asymptomatic infections and detects infections up to 34 months post-infection (25), hence it is a more accurate proxy of the cumulative number of infections that occurred in the population and reduces the risk of misclassification bias.

According to the current study, there is an overall higher number of infections among individuals working from home, however, our study design does not allow us to state that working from home has a causal effect on the risk of being infected. Importantly, our results should be interpreted in the context of a multinational company deciding to implement a specialized occupational safety unit that immediately deployed a wide range of sanitary measures from the beginning of the pandemic. Thus, it is likely that SARS-CoV-2 infections were more frequent outside the workplace (8, 26) and that implementation of a combination of measures, such as reducing work-related close contact, likely played an important role in SARS-CoV-2 transmission (3, 4, 16). Overall, this may explain why the seroprevalence in this sample was similar to that of the general working-age population of the cantons of Vaud (25%) and Fribourg (18%), where antibodies were measured during the same period (27).

Beyond the viral spread, the impact on individuals and the society must also be considered when implementing work from home (28). Mandatory working from home may have negative consequences on physical and mental health due to stress, anxiety, difficulty managing work-life balance, and social isolation (1, 28–30). However, it also allows for flexible work models, whereby employees can benefit from personalizing their work arrangements (31–33).

In this study, we found that participants employed in a multinational company, who worked most of the time from home during 2020, had higher odds to be infected with SARS-CoV-2 by February 2021 compared to those working on site with strict sanitary measures. In conclusion, our results suggest that, in a context of strict sanitary measures implemented in the workplace, employees working from home did not seem to be at lower risk of infection compared to those working on site, especially if living alone or with one other person.

## Data availability statement

The raw data supporting the conclusions of this article will be made available by the authors, without undue reservation.

## Ethics statement

The studies involving human participants were reviewed and approved by Cantonal Research Ethics Commission of Zürich and Vaud, Switzerland (BASEC 2020-01247). The patients/participants provided their written informed consent to participate in this study.

## Author contributions

P-YR, AC, and AS contributed to the design of the Corona Immunitas Nestlé study based on the protocol provided by the National Corona Immunitas team. AS and NP contributed to the data collection. P-YR, AC, DA, and AS contributed to the conception and design of the work. AS and DA drafted the manuscript and contributed to the statistical analysis and interpretation of data. DA was involved in supervision and support with data management. All authors contributed to the article and approved the submitted version.

## Conflict of interest

Authors IB-F, NP, and SCM were employed by Nestlé Research. The funder was involved in the recruitment and blood sample acquisition, but had no role in the design of the study, data analyses and interpretation of data. The funder provided suggestions on a draft of the manuscript, but the final decision on the content was made solely by the senior author P-YR. The remaining authors declare that the research was conducted in the absence of any commercial or financial relationships that could be construed as a potential conflict of interest.

## Publisher's note

All claims expressed in this article are solely those of the authors and do not necessarily represent those of their affiliated organizations, or those of the publisher, the editors and the reviewers. Any product that may be evaluated in this article, or claim that may be made by its manufacturer, is not guaranteed or endorsed by the publisher.
