# Supplementary material for: SARS-CoV-2 infection among employees working from home and on site: An occupational study in Switzerland
Source: Front Public Health. 2022 Sep 16;10:980482. doi: 10.3389/fpubh.2022.980482 (PMC9523570; doi:10.3389/fpubh.2022.980482)
Supplement: Supplementary file 1 [file Data_Sheet_1.PDF]

Supplementary Figure 1. Directed acyclic graph (DAG) used to identify potential confounders of the relationship between working from home and SARS-CoV-2 infection.

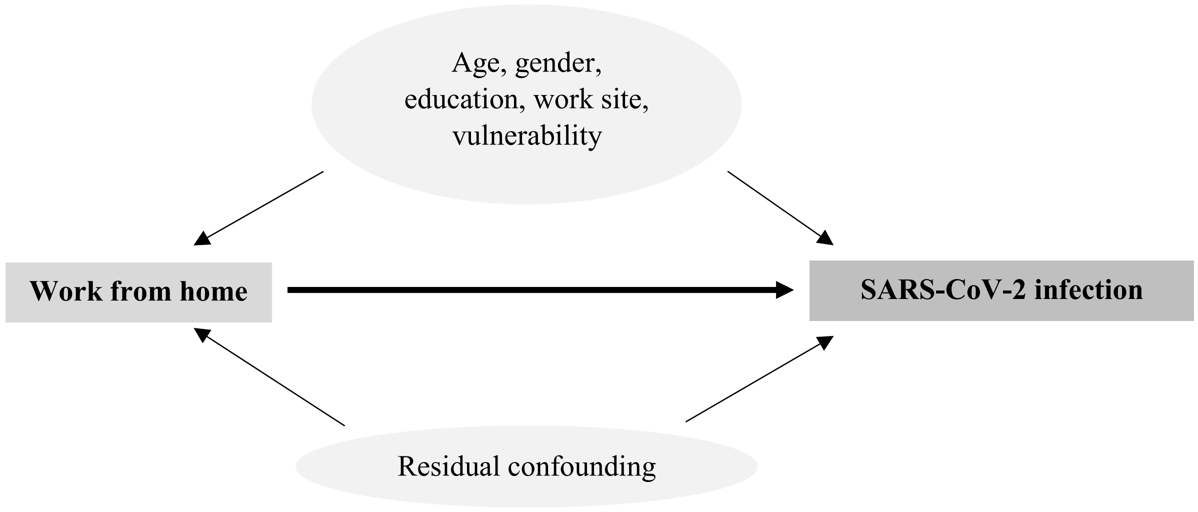

Supplementary Table 1. Sensitivity analysis - Association between work from home and SARS-CoV-2 infection assessed through logistic regression excluding participants who *only sometimes worked from home* (n=285).

	**Unadjusted OR**	**Adjusted OR***
**Work from home**		
Never	Ref	Ref
Most of the time	1.51 (0.61-2.21)	1.24 (0.65-2.42)

Number of participants never working from home = 126 (30%) among those 19 (15%) were infected with SARS-CoV-2; number of participants working most of the time from home = 159 (37%) among those 27 (17%) were infected with SARS-CoV-2.

* Adjusted for age and gender

Supplementary Table 2. Number of participants being infected or not with SARS-CoV-2 in each subgroup of potential effect modifiers.

	Infected with SARS-CoV-2	Not infected with SARS-CoV-2	Total
**Household size**			
Living alone or with 1 other person			
Working most of the time from home	16	50	66
Never or only sometimes working from home	13	100	113
Living with ≥ 2 other persons			
Working most of the time from home	11	82	93
Never or only sometimes working from home	26	127	153
**Children in household**			
Living without children			
Working most of the time from home	16	63	79
Never or only sometimes working from home	23	128	151
Living with ≥ 1 child			
Working most of the time from home	11	69	80
Never or only sometimes working from home	16	99	115
**Vulnerability criteria**			
None			
Working most of the time from home	23	101	124
Never or only sometimes working from home	30	183	213

≥ 1			
Working most of the time from home	4	31	35
Never or only sometimes working from home	9	44	53
**Worried about being infected**			
Not at all to moderately			
Working most of the time from home	23	117	140
Never or only sometimes working from home	31	181	212
Very to extremely			
Working most of the time from home	4	15	19
Never or only sometimes working from home	8	46	54
**Worried about adverse health consequences if infected**			
Not at all to moderately			
Working most of the time from home	19	114	133
Never or only sometimes working from home	32	184	216
Very to extremely			
Working most of the time from home	8	18	26
Never or only sometimes working from home	7	42	49
